# Factors Associated with Improved Knowledge of Metabolic Syndrome in Female Market Traders

**DOI:** 10.3390/ijerph191912256

**Published:** 2022-09-27

**Authors:** Gloria Achempim-Ansong, Amme M. Tshabalala, Philippe J. Gradidge

**Affiliations:** 1 Department of Nursing Education, Faculty of Health Sciences, University of the Witwatersrand, Johannesburg 2193, South Africa; 2 Centre for Exercise Science and Sports Medicine, Faculty of Health Sciences, University of the Witwatersrand, Johannesburg 2193, South Africa

**Keywords:** metabolic syndrome, market traders, knowledge, women’s health

## Abstract

Metabolic syndrome (MetS) is considered to be a clustering of cardiometabolic diseases and is emerging as a public health concern. There is little evidence of this disease in market traders, and so the aim of this study was to determine the prevalence and knowledge of MetS. In this cross-sectional study, anthropometry, blood pressure and bloods were collected using standardized methods to detect the prevalence of MetS using the harmonized method in a cohort of female Ghanaian market traders (n = 338). A questionnaire documented the knowledge of MetS. Linear regression was used to investigate the factors associated with knowledge and was reported as adjusted β values. Forty-two percent (n = 142) had MetS. The overall knowledge of MetS was low, driven by education (β = 0.22, *p* = 0.0001), low levels of high-density lipoprotein-cholesterol (β = −0.15, *p* = 0.018) and affiliation with the Ewe cultural group (β = −0.19, *p* = 0.0004). As females working in a sedentary occupation, market traders are vulnerable to MetS. Our findings indicate the urgent need for culturally sensitive education to promote healthy behaviours.

## 1. Introduction

Metabolic syndrome is a clustering of cardiometabolic diseases [1], and diagnosis is made when 3 out of the 5 cardiometabolic disease risk factors are present, including hyperglycaemia, hypertriglyceridemia, low HDL, central obesity and elevated blood pressure [2]. It is emerging as a public health concern in sub-Saharan African countries, especially amongst countries with a high prevalence of obesity [3]. The disease is associated with an increased risk of cardiovascular diseases, type 2 diabetes, stroke and coronary artery disease [4]. Women and the urban poor in sub-Saharan African countries have the highest prevalence of obesity and are therefore most vulnerable to MetS [3]. African diaspora is known to have a high prevalence of MetS compared with other ethnic groups, despite residing in high-income countries, and this may indicate a genetic predisposition to the disease [5].

The obesity epidemic is at the core of the aetiology of MetS [1], indicating an association with obesogenic environments and sedentary behaviour. The Republic of Ghana is an example of a low-income sub-Saharan African country experiencing rapid urbanization and the emergence of obesity-related chronic diseases in the adult population, but significantly higher in females than males [6]. As a result of the high levels of overweight and obesity, females have a higher prevalence of MetS than males [7], even amongst those previously considered as apparently healthy [8]. The increasing adoption of sedentary lifestyle and increasing weight gain with subsequent pregnancies are amongst the factors predisposing Ghanian women to MetS [9]. Recent evidence has shown that Ghanaians have a high prevalence of insufficient physical inactivity, independent of living in HICs versus LMICs [10]. Evidence from Ghana and other sub-Saharan African countries suggests prevention of disease through lifestyle modification that includes weight loss, physical activity and eating a healthy diet [11].

Having knowledge of disease and lifestyle behaviours to prevent the onset are important for public health strategies focussed on improving community health in African populations [12]. However, it is unknown whether the Ghanian population have an appreciation of MetS, its components and the risk factors associated with the disease. An important section of the population is Ghanaian market traders, who by design are sedentary for most of the day and highly exposed to an obesogenic environment, and despite being aware of policy regarding unhealthy meats [13] are still experiencing an increase in obesity [6]. Chen, Chiu and Chen [14] reported that non-sedentary workers aged ≤ 40 years were at lower risk of MetS than older workers (aged >60 years) in sedentary occupations (*p* < 0.05). Consequently, knowledge of MetS may influence their choices regarding lifestyle modifications to prevent the onset of disease. In light of this background, there were two main study aims: (a) to determine the level of MetS in a cohort of female Ghanaian market traders in Kaneshie, Accra and (b) to determine their knowledge of the disease.

## 2. Materials and Methods

This descriptive cross-sectional survey was conducted among women trading in Kaneshie, one of the ten large markets in Accra-Ghana. Workers are assumed to be sedentary, trading in relatively inactive vocations [15] and consuming highly processed foods [16] during the daily operational hours of the market, from 5:30 a.m. to 7 p.m. The study was approved by the Human Research Ethics Committee of the University of the Witwatersrand, South Africa (M170371) as well as the Institutional Review Board of the Noguchi Memorial Institute for Medical Research, University of Ghana (NMIMR-IRB CPN 088/16-17). Participants provided written informed consent.

Knowledge of MetS was assessed using a 9-item Knowledge of MetS (K-MS) questionnaire [15]. The questions were designed to determine the knowledge on the definition and detection of MetS, complications associated with MetS, prevention of MetS, and approaches to treatment and management of MetS. Each of the 9 questions had 5 options, one was correct and an additional option to indicate that the participant did not know the answer. The scores ranged from 0 to a total of 90, with correct scores allocated a ‘10’, and incorrect and ‘did not know’ answers allocated a ‘0’. Scores closer to 90 indicated a better understanding of MetS. The questionnaire was translated from the original Chinese into Twi, a common Ghanaian language to suit the context. Reverse translation into English was conducted to ensure face validity.

Participants wore minimal clothing with no shoes for all anthropometric measurements. An electronic digital scale with stadiometer was used for determining the weight (kg) and height (m) of participants (Adam Equipment Co, Milton Keynes, UK). Body mass index (BMI, kg/m^2^) was calculated and classified as normal (≥18.5 and <25 kg/m^2^), overweight (≥25 and <30 kg/m^2^) or obese (≥30 kg/m^2^). Waist circumference was measured with a flexible, non-elastic tape measure, at the narrowest part of the trunk, horizontally, while the participants were standing with arms at the side, a relaxed abdomen and feet together.

Blood pressure was measured with participants in a rested seated position and recorded manually using a standard sphygmomanometer. The mean of three readings at an interval of 5 minutes to the nearest mmHg. Fasting blood samples were collected using a point-of-care glucometer (Accu-Chek, Roche Diabetes Care GmbH, Mannheim, Germany). Blood specimens were taken from the participants using sterile syringes and needles by experienced and qualified laboratory technicians, for laboratory analysis of triglycerides and HDL.

Metabolic syndrome was defined using the harmonized criteria [2]. Thus, participants were diagnosed with MetS if any 3 of the following criteria were present, waist circumference ≥ 80cm, elevated triglycerides ≥ 1.7 mmol/L, reduced HDL < 1.3 mmol/L, elevated blood pressure systolic ≥ 130 and/or diastolic ≥ 85 mmHg, and elevated blood glucose ≥ 5.6 mmol/L [2].

Data analyses were performed using Statistica (version 13, StatSoft, Tulsa, OK, USA). Continuous variables are presented in tables as mean ± SD. Metabolic and anthropometric differences for metabolic knowledge scores were compared between groups using either a student’s unpaired t-test or analysis of variance. Multivariable linear regression analysis was performed to identify the factors associated with knowledge of MetS. The variables that were associated with knowledge of MetS score in the univariate analysis with *p* < 0.20 were all included in a single multivariable linear regression model with knowledge again as the outcome variable. Collinearity within this model was quantified via variance inflation factor (VIF) analysis, but none was observed with all VIFs < 3.00. We conducted univariate logistic regression analysis to determine the factors associated with the cardiometabolic components of MetS. These variables included tertiles of age, education status (completion of high school education or no completion of high school), marital status (single of living together), waist, knowledge of MetS and ethic group (Akan, Ewe, GA). All independent variables with a *p* < 0.20 from the univariate analysis were included in the multivariable logistic regression models with the cardiometabolic factors as the outcome variables.

## 3. Results

### 3.1. Subject Characteristics

Demographic, anthropometric and cardiometabolic characteristics of the market women participating in the study are shown in (Table 1). The mean age was 46.4 ± 10.6 years with the age range of 25 to 65 years. The majority (53.6%) were living with a partner, were affiliated with the Akan culture (59.2%) and had incomplete or no formal schooling (75.1%). A lower number of participants self-reported a history of hypertension (24.6%) or diabetes (6.5%). Most participants were classified as overweight or obese (80.8%) using the BMI categories. High serum triglycerides were the lowest ranking risk factors, followed by hyperglycaemia (22.5%), elevated BP (49.7%), low HDL (68.9%) and central obesity (88.2%), in fourth, third, second and first place, respectively. The presence of MetS was indicated by 42% having at least three of the risk factors.

### 3.2. Presence of Cardiometabolic Disease Risk Factors

The BMI (30.7 ± 6.5 kg/m^2^) and waist circumference (95.3 ± 12.9 cm) shown in Table 2 are consistent with the data in Table 1, suggesting a high presence of obesity. The systolic BP (128.2 ± 22.1 mmHg) and diastolic BP (77.1 ± 12.8 mmHg) levels are close to the cut-off for the diagnosis of MetS. The triglycerides (1.0 ± 0.3 mmol/L) and fasting glucose levels (5.4 ± 2.4 mmol/L) are within the normal ranges; however, the HDL levels (1.2 ± 0.3 mmol/L) are lower than the cut-point for defining MetS.

### 3.3. Factors Associated with Knowledge of MetS

The MetS knowledge of the participants is presented in Table 3, displayed by individual items. The mean total score for the scale was 42.2 ± 11.6, with a potential score ranging from 0 to 90. The participants had 3.2 correct responses on average for the scale ac participants had no knowledge for 5.2 of the items. The item on the medical management of MetS had the most correct answers (96.4%), followed by the item pertaining to behaviours associated with MetS (94.4%). Items 1 to 4, pertaining to the definition of MetS, have the least correct answers. The item with the most participants (99.1%) having no knowledge was item 5, indicating no knowledge of MetS complications.

**Figure 1:**
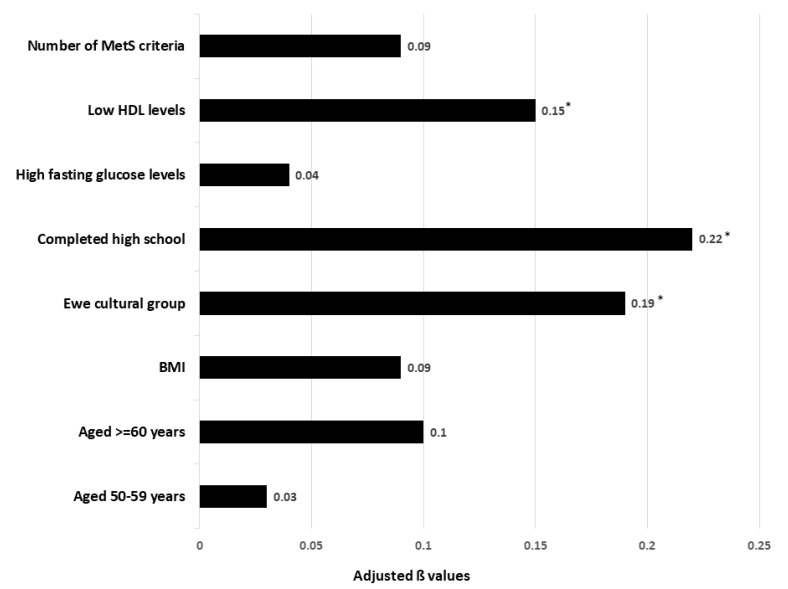
Factors associated with MetS knowledge (n = 338); F (8.329) = 6.3270, adjusted R^2^ = 0.112 *p* < 0.0001. BMI = body mass index; HDL = high density lipoprotein cholesterol; * *p* < 0.05.

Age was not correlated with MetS knowledge (r = −0.02, *p* = 0.722), but there were differences for age categories (*p* = 0.012, Table 1). The bivariate analysis shows that participants with different cultures (*p* = 0.011), years of education (*p* = 0.000), marital status (*p* = 0.035) and hyperglycaemia (*p* = 0.041) had different MetS knowledge scores. A multivariable linear regression model was conducted to determine the factors associated with MetS knowledge (Figure 1). The final regression model revealed that knowledge of MetS was associated with low HDL, completion of high school, and being affiliated with the Ewe cultural group. The variation inflation factors were <1.0, indicating no multicollinearity amongst the variables. These factors also explained 11.2% of the variation in the knowledge of MetS (*p* < 0.0001).

**Figure 2:**
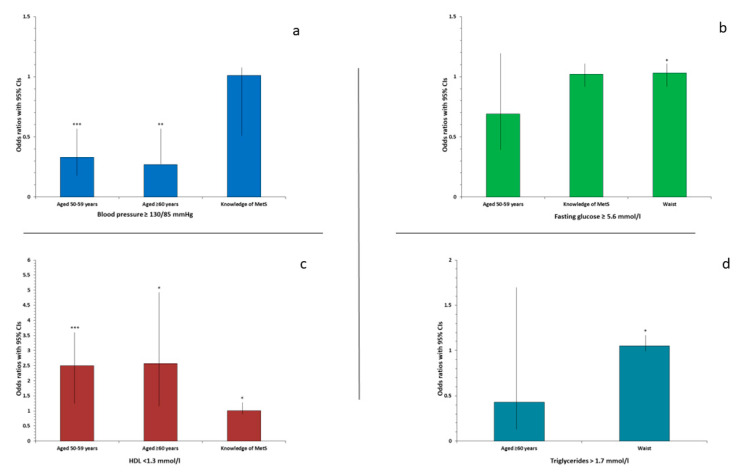
Risk factors associated with cardiometabolic components of MetS. (**a**). Blood pressure ≥ 130/85 mmHg, (**b**). Fasting blood glucose ≥ 5.6 mmol/L, (**c**). High density lipoprotein cholesterol (HDL) < 1.3 mmol/L, (**d**). Triglyceride levels > 1.7 mmol/l; * *p* < 0.05, ** *p* < 0.005, *** *p* < 0.0005.

The independent variables with *p* < 0.20 from univariate logistic regression were included in the multivariable logistic regression analysis for elevated blood pressure, elevate glucose levels, elevate triglyceride levels, low HDL levels as dependent variables, respectively (Figure 2). An age of 50+ is associated with an increased risk of low HDL levels, whilst having a better knowledge of MetS protects against low HDL levels. Waist is associated with higher risk of high fasting glucose levels and high triglyceride levels, whilst an age of 50+ protects against elevated blood pressure.

## 4. Discussion

The purpose of this cross-sectional study was to determine the prevalence of MetS of a sample of females working at an established marketplace in Accra, Republic of Ghana and to examine their knowledge of the disease. Our results show that 42% of the participants had at least three of the five criteria for MetS. This prevalence is higher than most HICs, but similar to other sub-Saharan African countries [3]. Most importantly, this is the first study that has investigated the knowledge of MetS of market traders. The novel finding is that the low knowledge of MetS was associated with increased risk of low levels of HDL. The only items correctly reported by most of the participants concerned knowledge of central adiposity, lifestyle behaviours, preventative measures and medical management of MetS. The most significant contributors to better knowledge scores were completion of high school, while lower knowledge was associated with poor lipid management and affiliation with the Ewe cultural group. As expected, additional analysis shows that waist, a proxy measure of central fat, is positively associated with elevated fasting glucose and high triglyceride levels [17]. The finding of a negative association between knowledge of MetS and low HDL levels supports the notion of developing initiatives to inform this study population on the risk factors associated with MetS.

The mean knowledge score of MetS was 44.2 out of a possible score of 100. Eighty percent (n = 270) of the participants had knowledge scores less than 50 out of 100, indicating that the majority had poor knowledge levels, which are consistent with population groups from India [18], Jordan [19], Xian, Mainland China [20] and Hong Kong, China [21]. A study conducted in Sri Lanka reported a higher knowledge of MetS in most of the study population, but still struggled to answer questions regarding lifestyle preventative measures correctly [22]. For instance, most of the Sri Lankan group (97%) were unaware of the normal BMI range, i.e., values ranging from 18.5 to 24.9 kg/m^2^ for adult populations and this was despite being aware of individual cardiovascular diseases risk factors. A study conducted in Western Turkey reported even lower knowledge of MetS compared with our data [23]. In comparison, our study findings show that study participants had difficulty with recognizing components of MetS but were competent with behavioural modifications to reduce risk, and this is reason for public health initiatives to improve awareness of disease in this population.

As observed in a recent systematic review [17], evidence indicates that the prevalence of metabolic in sub-Saharan African countries, varies by region with Southern African countries reporting higher prevalence compared with Northern, Eastern and Western regions. The Republic of Ghana has an estimated prevalence of MetS ranging from ~12% to 46%, indicating that our study participants fall into the higher range. However, only 13% of our study population correctly answered items concerning the definition of MetS, and this is consistent with the Xian, Mainland China [20]. As observed in the Sri Lankan study, an adequate awareness of disease is required for participants to fully comprehend the presence of MetS and the increased cardiovascular diseases risk [22]. In contrast, a cohort of students from Michigan University in the United States demonstrated better knowledge of MetS and related complications [24]. These students were clearly younger than our study population and more aware of cardiovascular disease risk factors; however, the data are self-reported, indicating the need for more robust research. Our study findings agree with the fact that education contributes to better knowledge MetS, independent of socioeconomic region [25,26].

One striking observation from our study findings is that only 2.7% of the participants knew the criteria for the central obesity. In sub-Saharan African countries, the female waist circumference cut-off for detecting MetS is currently 80 cm, but more recent data suggest a higher threshold more aligned with the male cut-off of 94 cm [27]. Our study findings are comparable to the data from Xian, China [20], demonstrating that only a few participants were familiar with the diagnostic criteria for elevated blood pressure (16.9%) and hyperglycaemia (6.2%), but had inadequate lipid management. We postulated that these findings could be attributed to possible mechanisms. First, the education and healthcare system in the Republic of Ghana has experienced challenges in health literacy despite a policy of universal healthcare [28]. Second, the West African region is experiencing an increasing burden of non-communicable diseases not dissimilar to other sub-Saharan African countries, and this is due to rapid urbanization and an increasing prevalence of obesity [6]. Finally, there does not seem to be cohesion amongst initiatives to reduce the prevalence of non-communicable diseases [29]. Accordingly, we propose that policy makers introduce health education from formative schooling years. Indeed, evidence suggests that knowledge of chronic conditions amongst Ghanaian healthcare requires attention [28].

Intermittent non-sedentary activities during vocational hours can aid in breaking up extended sitting time and reduce the risk of MetS [14]. Our study findings provide important developmental data for occupational healthcare providers to develop workplace interventions for at risk workers in this setting. The workers should be included in the wide spectrum of health transforming activities as they are integral to success of future strategies. Future research is required to developing linkage of those persons with MetS to healthcare at the Kaneshie Clinic, a healthcare clinic located close to the Accra marketplace. Healthcare providers from this clinic need to develop screening and rapport with market traders onsite to ensure sustainability of initiatives, considering low resources

A key limitation of this study was that it only included market traders from one marketplace in Accra and therefore cannot be generalized to the rest of the Republic of Ghana. The women were assumed to be sedentary because of the nature of their work, however sedentary needs to be collected using self-reporting instruments or objective triaxial data. Financial constraints however limit the use of specialized tools in this setting. Finally, only 11% of the variance in knowledge of MetS could be explained by the independent factors, indicating that further investigations consider whether other factors such as lifestyle and environmental factors (e.g., dietary pattern, physical activity and smoking) are associated with knowledge of MetS and the cardiometabolic components of MetS. This would help to develop more tailored interventions for the study population.

## 5. Conclusions

In summary, our study findings indicate that the prevalence of MetS in this cohort of female Ghanaian market traders was high, but similar to other African populations in the region. Our study findings demonstrate that this is driven in part by a poor knowledge of MetS as observed in those individuals with lower educational attainment. These workers could clearly benefit from public health interventions to improve the knowledge and prevention of cardiometabolic diseases associated with MetS. These initiatives could include initiatives to interrupt extended sitting during vocational hours. Certainly, global physical activity policy recommends breaking up sedentary behaviour to improve cardiometabolic health.

## Figures and Tables

**Table 1:** Demographic, anthropometric, cardiometabolic characteristics and knowledge of MetS.

Variables	MetS Knowledge Score	*t*-Test or ANOVA *p*-Value for Model
Age categories (years)		0.012
25–39 (n = 98)	42.0 ± 11.1	
40–49 (n = 96)	42.5 ± 11.7	
50–59 (n = 103)	43.5 ± 11.4	
≥60 (n = 41)	39.0 ± 12.7	
Marital status		0.035
Widowed, divorced or single (n = 157)	41.4 ± 11.7	
Living together (n = 181)	42.9 ± 11.5	
Highest level of education		0.000
None (n = 39)	37.0 ± 10.3	
Primary school (n = 52)	40.2 ± 11.7	
Middle school (n = 163)	41.9 ± 11.2	
Secondary school (n = 72)	46.9 ± 11.5	
Tertiary education (n = 12)	44.4 ± 12.5	
Cultural affiliation		0.011
Akan (n = 200)	42.3 ± 12.3	
Ewe (n = 17)	33.3 ± 14.2	
GA (n = 91)	43.0 ± 9.5	
Other (n = 30)	44.1 ± 9.5	
Self-reported hypertension		0.098
Yes (n = 83)	41.6 ± 11.2	
No (n = 255)	44.0 ± 12.8	
Self-reported diabetes		0.741
Yes (n = 22)	41.1 ± 13.8	
No (n = 316)	42.3 ± 11.5	
BMI categories		0.069
<25 kg/m^2^ (n = 65)	39.5 ± 9.2	
25–29.9 kg/m^2^ (n = 105)	42.0 ± 10.8	
≥30 kg/m^2^ (n = 168)	43.4 ± 12.8	
Central fat using waist		0.361
≥80 cm (n = 298)	42.2 ± 11.8	
<80 cm (n = 40)	39.2 ± 9.7	
Elevated BP		0.956
Yes (n = 168)	43.0 ± 11.9	
No (n = 170)	41.4 ± 11.3	
Fasting glucose ≥ 5.6 mmol/L		0.041
Yes (n = 76)	44.9 ± 13.1	
No (n = 262)	41.4 ± 11.0	
Serum triglycerides ≥ 1.7 mmol/L		0.177
Yes (n = 10)	46.7 ± 4.7	
No (n = 328)	42.1 ± 11.7	
HDL < 1.3 mmol/L		0.202
Yes (n = 233)	41.1 ± 11.3	
No (n = 105)	44.8 ± 12.0	
Number of MetS risk factors		0.529
0 (n = 1)	44.4 ± 0	
1 (n = 50)	39.6 ± 10.3	
2 (n = 145)	42.5 ± 11.2	
3 (n = 123)	42.9 ± 12.8	
4 (n = 19)	42.7 ± 10.0	
Presence of MetS		0.615
Yes (n = 142)	42.9 ± 12.4	
No (n = 196)	41.7 ± 11.0	

Data expressed as mean ± SD; BMI = body mass index; BP = blood pressure; HDL = high density lipoprotein cholesterol; MetS, metabolic syndrome; WC = waist circumference.

**Table 2:** Presence of anthropometric and cardiometabolic risk factors.

Risk Factors	Mean ± SD
BMI (kg/m^2^)	30.7 ± 6.5
WC (cm)	95.3 ± 12.9
Total cholesterol (mmol/L)	5.5 ± 1.1
Triglycerides (mmol/L)	1.0 ± 0.3
HDL (mmol/L)	1.2 ± 0.3
Systolic BP (mm Hg)	128.2 ± 22.1
Diastolic BP (mm Hg)	77.1 ± 12.8
Blood glucose (mmol/L)	5.4 ± 2.4

Data expressed as mean ± SD; HDL = high density lipoprotein cholesterol; WC = waist circumference; BMI = body mass index; BP = blood pressure.

**Table 3:** MetS knowledge scores by scale item.

Item Number	Knowledge Criteria	Mean ± SD	Correct Answer n (%)	Do Not Know n (%)
1	Definition of MetS?	0.13 ± 0.34	44 (13)	294 (87)
2	Waist circumference (≥80 cm)	0.03 ± 0.16	9 (2.7)	329 (97.3)
3	Elevated blood pressure (Systolic ≥130 and/or diastolic ≥ 85 mm Hg)	0.17 ± 0.38	57 (16.9)	281 (83.1)
4	Fasting glucose (≥5.6 mmol/L)	0.06 ± 0.24	21 (6.2)	317 (93.8)
5	Complications of MetS	0.01 ± 0.1	3 (0.9)	335 (99.1)
6	Consequences of waist ≥ 80 cm	0.66 ± 0.47	224 (66.3)	114 (33.7)
7	Behaviours associated with MetS	0.94 ± 0.23	319 (94.4)	19 (5.6)
8	Self-care once diagnosed with MetS	0.83 ± 0.37	281 (83.1)	57 (16.9)
9	Medical treatment of MetS	0.96 ± 0.19	326 (96.4)	12 (3.6)
	Total questionnaire score range (0–90)	42.2 ± 11.6	1284 (37.9)	1785 (52.0)

## Data Availability

Anonymized study data are available upon motivated and reasonable request to the corresponding author.
